# Post COVID-19 condition among adults in Malaysia following the Omicron wave: A prospective cohort study

**DOI:** 10.1371/journal.pone.0296488

**Published:** 2024-01-05

**Authors:** Peter Seah Keng Tok, Kong Yeow Kang, Sock Wen Ng, Norazida Ab Rahman, Muhammad Aminul Syahmi, Mohan Dass Pathmanathan, Maheshwara Rao Appannan, Kalaiarasu M. Peariasamy, Sheamini Sivasampu

**Affiliations:** 1 Institute for Clinical Research, National Institutes of Health, Ministry of Health Malaysia, Shah Alam, Selangor, Malaysia; 2 Hospital Sultanah Bahiyah, Ministry of Health Malaysia, Alor Setar, Kedah, Malaysia; 3 Disease Control Division, Ministry of Health Malaysia, Putrajaya, Malaysia; Korea Disease Control and Prevention Agency, REPUBLIC OF KOREA

## Abstract

Post COVID-19 condition is an important public health problem as we emerge from the COVID-19 pandemic. In this prospective cohort study, we aimed to determine the prevalence of this condition and assess its associated factors and impact on health-related quality of life in a population setting in Malaysia. Study was conducted from April to June 2022 when the Omicron variant predominated. All individuals testing positive for SARS-CoV-2 infection (RT-PCR, RTK-Ag) were invited for participation. Study questionnaires were delivered via the *MySejahtera* platform (mobile application). From the total of 44,386 participants who provided responses up to 3-months interval, 1,510 participants (3.4%) fulfilled the post COVID-19 condition criteria. Majority of the affected participants (83.8%, n = 1,265) experienced either cough, fatigue or forgetfulness–the three most common symptoms. Being females, having existing comorbidities, presence of symptoms and requiring hospital admission during the acute illness were associated with higher likelihoods of developing the post COVID-19 condition at 3-months interval. Amongst the 1,510 individuals, one in five had limitations in performing their usual daily activities while at least one in three expressed that their work was affected. Understanding this condition better is essential to guide strategic and responsive plans of action, which may require coordinated multidisciplinary interventions.

## Introduction

Post COVID-19 condition can affect anyone exposed to SARS-CoV-2 infection, regardless of their age and severity of the acute illness [1–3]. Typically, the condition affects multiple organ systems including but not limited to: respiratory, cardiovascular, neurological, gastrointestinal, dermatologic, and musculoskeletal systems [1, 2, 4–11]. Affected individuals have reported symptoms including fatigue or muscle weakness, dyspnea, sleep disturbances, cognitive impairment, symptoms of post-traumatic stress disorder, mental health disorders, headache, arthralgia and myalgia. Psychological and social impacts have also been reported, including on health-related quality of life [12–14].

This persistence of symptoms in some individuals following coronavirus disease 2019 (COVID-19) was referred to by several common terms including long COVID, long haulers, post-acute COVID-19 syndrome, post-acute sequelae of COVID-19 (PASC), and post COVID-19 condition [15–19]. The World Health Organization (WHO) using the Delphi consensus methodology recently developed a clinical case condition for this “post COVID-19 condition”, defined as the continuation or development of new symptoms three months after the initial SARS-CoV-2 infection, with these symptoms lasting for at least two months with no other explanation [3, 19].

Existing studies on this condition mostly involved hospitalised individuals (more severe forms of acute COVID-19) and earlier variants of SARS-CoV-2, particularly the Delta variant [9, 20]. A meta-analysis of 41 studies has estimated the pooled prevalence of this condition at 43% (95% CI 39%, 46%) [21], but most of the studies included preceded the Omicron variant surge. This gap, together with studies demonstrating the impact of COVID-19 vaccination on post COVID-19 condition [2, 22, 23], necessitates further assessment of this condition given the overwhelming proportion of recent SARS-CoV-2 infections contributed by the Omicron variant and the increasingly vaccinated populations against COVID-19 globally. This is also especially important for low- and middle-income countries (LMICs), where reported studies are notably scarce [9].

Malaysia is an upper-middle-income country with an estimated 32.9 million population in 2022 [24]. More than 95% of the adult population in Malaysia were fully vaccinated against COVID-19 by the end of 2021 [25] but like many other countries globally, Malaysia was not spared by the Omicron variant onslaught. In January 2022, the Omicron variant had contributed to 80% of the samples sequenced under the genomic surveillance in Malaysia [26]. A surge of confirmed COVID-19 cases then ensued from February to March 2022, where over 20,000 cases/day were recorded on average [27]. Cumulatively, as of December 2022, over five million confirmed cases of SARS-CoV-2 infections have been documented in Malaysia [28].

Although the majority of the COVID-19 cases reported full clinical recovery, given the backdrop of an ageing society [24] and the significant burden of non-communicable diseases (NCDs) among the population in Malaysia, particularly for cardiovascular diseases, diabetes and cancer [29, 30], an assessment of the burden and impact of this post COVID-19 condition is warranted. The present study, therefore, aims to determine the prevalence of post COVID-19 condition in a population setting in Malaysia following the Omicron wave of SARS-CoV-2 infections, by using the recent WHO definition for this condition. We also aimed to assess the factors associated with the development of this condition and its impact on health-related quality of life amongst those affected.

## Materials and methods

### Study design and data collection

This was a prospective, observational cohort study conducted nationwide in Malaysia. All individuals aged 18 years and above who tested positive for SARS-CoV-2 infection between 23^rd^ April 2022 and 24^th^ June 2022, either via reverse-transcriptase polymerase chain reaction assay (RT-PCR) or rapid test kit for antigen testing (RTK-Ag), were invited for participation in the study. The diagnosis and testing did not include the type of variant, as routine genomic surveillance is not performed in Malaysia. In a previous study, 5^th^ February 2022 was estimated as the start of the pre-dominant Omicron period in Malaysia by using the Bai-Perron sequential breakpoint test [25] and therefore, the Omicron variant is presumed to be the predominant variant in circulation during this study.

All study participants provided voluntary informed consent before a self-reported online study questionnaire was administered seven days after the date of the positive test. This assessment at baseline constituted the acute phase of illness following the SARS-CoV-2 infection. Participants were then followed up at 1-month, 3-months and 6-months intervals, similarly via self-reported questionnaires, to assess for the continued presence of symptoms (if any) and its impact on health-related quality of life. The informed consent procedure, as well as all data collection in this study, were done electronically and utilised the *MySejahtera* platform (mobile application). *Mysejahtera* platform was developed to assist the management of the COVID-19 pandemic in Malaysia with functions including uploading and tracking of risk status, testing and results for SARS-CoV-2 infection, self-health assessment and status monitoring, as well as COVID-19 vaccination appointment and certificate [31]. The platform was available for free and was widely used by the Malaysian public.

### Study measures and operational definition

Study questionnaire was developed by adapting findings from a literature review on the frequent symptoms of COVID-19 and gathering consensus from local experts in infectious diseases, epidemiology, public health and clinical research. There were three main sections of the questionnaire: the first section collected demographic characteristics and underlying medical co-morbidities; the second section captured the presence of COVID-19 related symptoms, while the third section contained questions on the impact of COVID-19 on the health-related quality of life of the individuals–including on their mobility, self-care, activities of daily living, emotions and ability to (return to) work. Study questionnaire at baseline is provided in the S1 File.

In Malaysia, the clinical management of confirmed COVID-19 cases in adults follows the guidelines by the Ministry of Health Malaysia [32]. This included the consideration and criteria for hospital admission. Further details on the hospital admission, including the clinical staging of cases, are provided in the S2 File.

The definition of study outcome of interest, or “post COVID-19 condition”, adapts the clinical case definition developed by the WHO [19]. In this study, to fulfil the criteria of “post COVID-19 condition”, an individual must have had continuing symptoms or developed new symptoms three months after being diagnosed positive (RT-PCR or RTK-Ag) for SARS-CoV-2 infection, and the symptoms needed to last for at least two months duration.

### Statistical analysis

Demographic and clinical characteristics of participants were described as counts with percentages for categorical variables and means with standard deviations for continuous variables. Prevalence of post COVID-19 condition and prevalence of specific COVID-19 related symptoms were reported based on the number of participants with the condition during the follow-up period. McNemar’s test was used to compare health-related quality of life measures among individuals with post COVID-19 condition at baseline and after 3-months. Logistic regression was performed to identify factors potentially associated with post COVID-19 condition. The dependent variable was the presence of post COVID-19 condition. Independent variables were identified based on prior knowledge and clinical relevance. Univariate logistic regression analysis was conducted by including each variable separately in the models and variables with p-value less than 0.25 were entered into the multivariable model. Adjusted odds ratio and the corresponding 95% confidence intervals were estimated based on multivariable logistic regression model and correction for multiple testing by the Holm method. Variables were checked for multicollinearity and assumptions for the regression model. Statistical significance was set at p-value less than 0.05. All analyses were conducted using R version 4.1.2 in R Studio.

### Ethical consideration

Participants’ informed consent were obtained electronically via the *Mysejahtera* platform (mobile application). All data collected for the purpose of the study was deidentified. Study was registered in the National Medical Research Register (NMRR-21-880-59662) and ethical approval for study conduct was granted by the Medical Research and Ethics Committee (MREC), Ministry of Health, Malaysia.

## Results

**Fig 1:**
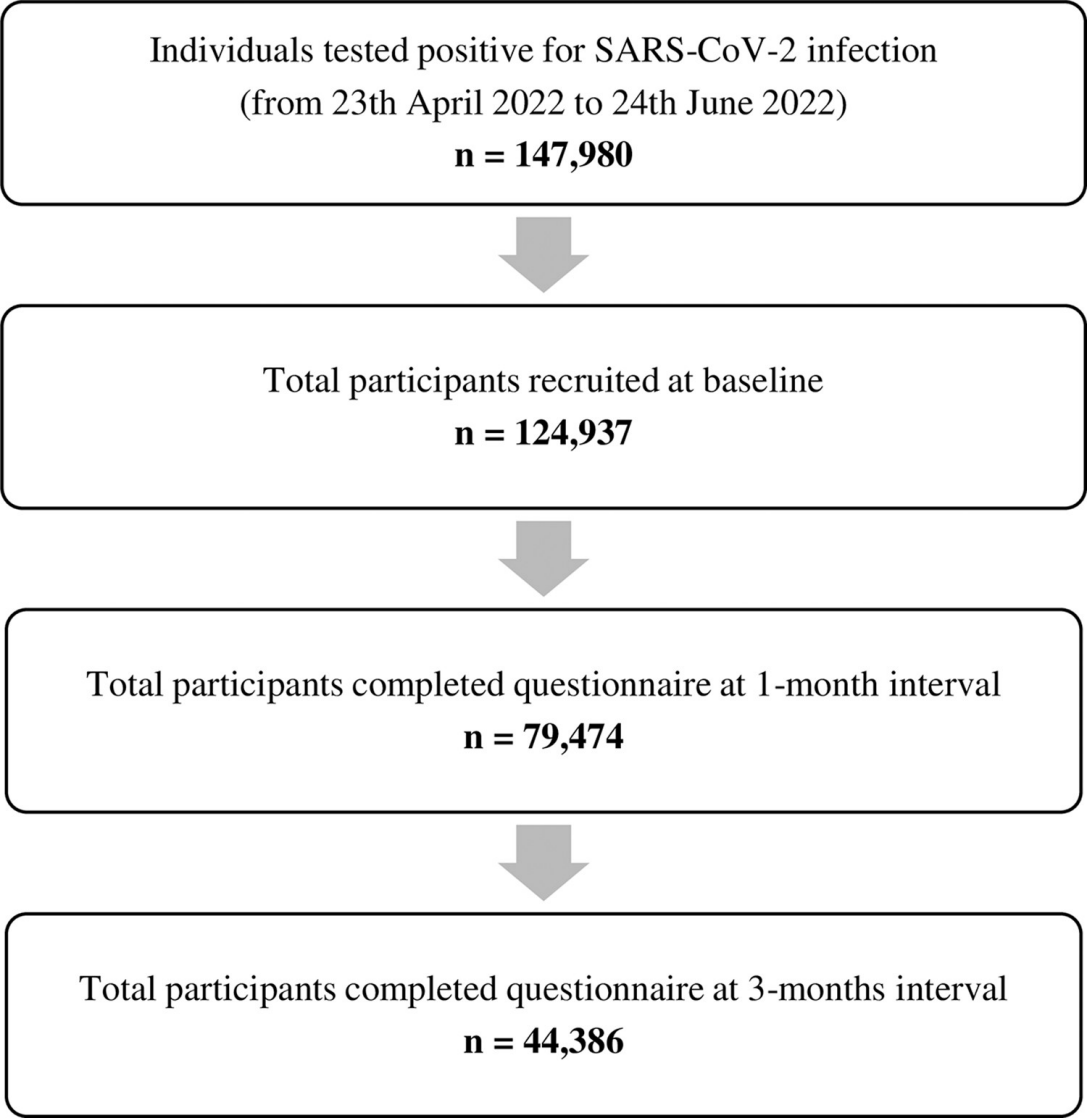
Study participants at data collection points throughout the study duration.

A total of 124,937 participants completed the study questionnaire during baseline (seven days after being diagnosed positive for SARS-CoV-2 infection). Subsequently, 79,474 participants (63.6%) responded during the 1-month interval and 44,386 participants (35.5%) responded during the 3-months interval, which were included in the final analysis (Fig 1).

Table 1 describes the socio-demographic and COVID-19 characteristics of all the participants. Amongst the 44,386 participants at 3-months follow-up included in the final analysis, the majority of them were females, aged between 30 and 59 years old, received at least secondary education and had been fully vaccinated against COVID-19. Medical comorbidities were present in around a quarter of the participants (25.7%, n = 11,422), with obesity, hypertension and diabetes mellitus being the most common. Within this cohort of 44,386 participants, 10,194 (24.6%) of them were symptomatic during the acute phase following SARS-CoV-2 infection while 1,088 (2.5%) of them were admitted to hospital, 268 (0.6%) needed supplemental oxygen and 122 (0.3%) required intensive care unit (ICU) admission.

**Table 1:** Socio-demographic and COVID-19 characteristics of study participants at baseline.

Characteristics	Patients at baseline		Patients with 3 months follow-up		Patients with Post COVID-19 condition	
	N = 124,937		N = 44,386		N = 1,510	
Age, years						
Mean (SD)	36.1	12.5	38.2	11.9	36.5	9.5
18–29	44,660	35.7%	11,699	26.4%	362	24.0%
30–59	73,647	58.9%	30,393	68.5%	1,121	74.2%
≥ 60	6,630	5.3%	2,294	5.2%	27	1.8%
Sex						
Female	73,778	59.1%	26,900	60.6%	1,171	77.5%
Male	51,159	40.9%	17,486	39.4%	339	22.5%
Ethnicity						
Malay	64,767	51.8%	22,030	49.6%	846	56.0%
Chinese	43,789	35.0%	17,302	39.0%	539	35.7%
Indian	9,202	7.4%	2,605	5.9%	68	4.5%
Others	7,179	5.7%	2,449	5.5%	57	3.8%
Educational level^†^						
Tertiary	87,301	69.9%	32,023	72.1%	1,291	85.5%
Secondary	32,943	26.4%	11,350	25.6%	210	13.9%
Primary	2,692	2.2%	639	1.4%	5	0.3%
No formal education	2,001	1.6%	374	0.8%	4	0.3%
Smoking status						
Smoker	13,382	10.7%	4,135	9.3%	70	4.6%
Non-smoker	111,555	89.3%	40,251	90.7%	1,440	95.4%
Comorbidities						
Present (at least one)	28,680	23.0%	11,422	25.7%	504	33.4%
Not present (no comorbid)	96,257	77.0%	32,964	74.3%	1,006	66.6%
Type of comorbidities						
Obesity	11,695	9.4%	4,713	10.6%	286	18.9%
Diabetes mellitus	8,515	6.8%	3,245	7.3%	92	6.1%
Hypertension	14,959	12.0%	6,029	13.6%	184	12.2%
Heart disease	3,243	2.6%	1,191	2.7%	21	1.4%
Kidney disease	1,922	1.5%	613	1.4%	9	0.6%
Lung disease	4,360	3.5%	1,677	3.8%	115	7.6%
Cancer	1,890	1.5%	666	1.5%	11	0.7%
COVID-19 vaccination status						
Boosted	121,129	97.0%	43,489	98.0%	1,462	96.8%
Fully vaccinated	3,792	3.0%	892	2.0%	48	3.2%
Not vaccinated/partially vaccinated	16	0.0%	5	0.0%	0	0.0%
Presence of symptoms during acute phase*						
Symptomatic	28,800	23.1%	10,941	24.6%	1,226	81.2%
Asymptomatic	96,137	76.9%	33,445	75.4%	284	18.8%
Clinical management during acute phase*						
Required hospital admission	2,057	1.6%	1,088	2.5%	73	4.8%
Required ICU admission	256	0.2%	122	0.3%	7	0.5%
Required supplemental oxygen	268	0.2%	268	0.6%	13	0.9%

^†^ Educational level: Primary, in general, refers to six years of primary school education (Standard 1 to 6, usually from age 6+ to 11+), Secondary refers to at least five years of secondary school education (Form 1 to 5, usually from age 12+ to 16+), while Tertiary refers to higher education in college or university.

*Acute phase refers to the period when the individuals were tested positive for COVID-19; a baseline assessment questionnaire was administered seven days after the positive test date for all participants.

Abbreviation: ICU, intensive care unit; NA, not applicable; SD, standard deviation

**Fig 2:**
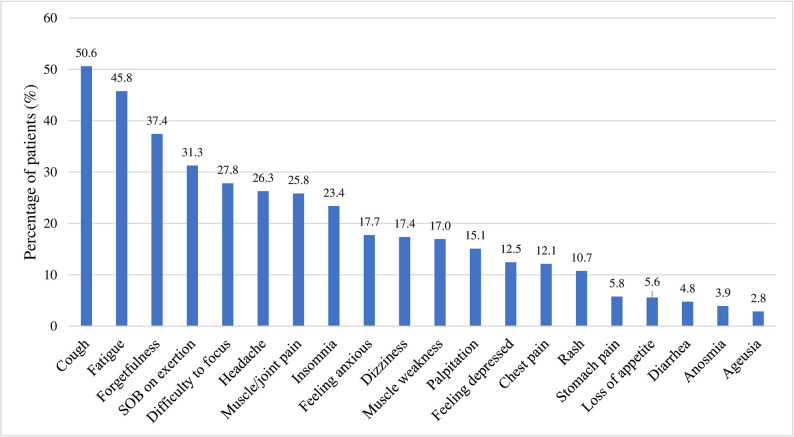
Distribution of symptoms at 3 months among study participants with post COVID-19 condition (n = 1,510). Abbreviation: SOB, shortness of breath.

At the 3-month interval, 1,510 participants or 3.4% of the total 44,386 participants reported persistence of COVID-19 related symptoms, thus fulfilling the post COVID-19 condition criteria. Fig 2 depicts the COVID-19 related symptoms and their corresponding prevalence among study participants. Among those who fulfilled the post COVID-19 condition criteria, the most commonly reported symptoms included cough (50.6%, n = 764), fatigue (45.8%, n = 691), forgetfulness (37.4%, n = 565), shortness of breath on exertion (31.3%, n = 472), difficulty to focus (27.8%, n = 420), headache (n = 26.5%, n = 397), muscle or joint pain (25.8%, n = 390) and insomnia (23.4%, n = 353). Majority of the participants (83.8%, n = 1,265) had either one of the three most common symptoms–cough, fatigue or forgetfulness. Other symptoms investigated and reported (<20% respectively) included feeling anxious, dizziness, muscle weakness, palpitation, feeling depressed, chest pain, skin rash, stomach pain, loss of appetite, diarrhea, anosmia and ageusia.

In the multivariable logistic regression (Table 2), age group 30–59 years old (compared to 18–29 years old), females (compared to males), presence of comorbidities, presence of symptoms and requiring hospital admission during the acute infection were found to have higher odds for the development of post COVID-19 condition. Being symptomatic during the acute infection was associated with an estimated 13 times higher odds (adjusted odds ratio 13.3, 95% CI 11.7, 15.2) higher odds for post COVID-19 condition. In contrast, the elderly age group (60 years and above) and being of other ethnicities had lower odds of developing the post COVID-19 condition. These factors remained significant after correction for multiple testing by the Holm method.

**Table 2:** Factors associated with development of post COVID-19 condition at 3 months interval.

Variable	Adjusted OR (95% CI)	p-value
Age		
18–29	Ref	
30–59	1.22 (1.08, 1.39)	0.002
≥60	0.50 (0.33, 0.74)	0.001
Sex		
Male	Ref	
Female	1.67 (1.41, 1.91)	<0.001
Ethnicity		
Malay	Ref	
Chinese	0.92 (0.82, 1.03)	0.154
Indian	0.93 (0.71, 1.19)	0.571
Others	0.69 (0.52, 0.91)	0.010
Smoking status		
Non-smoker	Ref	
Smoker	1.23 (0.96, 1.61)	0.101
Comorbidities		
Not present (no comorbid)	Ref	
Present (at least one)	1.40 (1.25, 1.58)	<0.001
Presence of symptoms during acute phase		
Asymptomatic	Ref	
Symptomatic	13.32 (11.69, 15.23)	<0.001
Hospital admission during acute phase		
No	Ref	
Yes	1.72 (1.32, 2.22)	<0.001

Abbreviation: OR, odds ratio; CI, confidence interval; Ref, reference

The final section of the study questionnaire captured the impact of post COVID-19 condition on the participants’ health-related quality of life (Table 3). At 3-months interval following SARS-CoV-2 infection, amongst the 1,510 participants who had post COVID-19 condition, 10.3% (n = 155) had symptoms limiting their mobility (walking), 1.5% (n = 33) had symptoms limiting their ability for self-care and hygiene, 22.1% (n = 333) had limitations in performing usual activities (housework, participation in family or leisure activities), 7.6% (n = 115) cannot live alone without any assistance from another person and 33.8% (n = 511) experienced symptoms that led to negative emotions such as sadness, anxiety and depression. Further, 54.0% (n = 816) of participants who had the condition reported that their work was unaffected at 3-months interval, 38.7% (n = 584) expressed that their work was affected but still able to continue working while 1.3% (n = 19) either lost their job or had to change job(s). Findings were largely similar to those reported at baseline during the acute phase following the SARS-CoV-2 infection, indicating that the impact lasted through for at least three months.

**Table 3:** Health-related quality of life findings for study participants.

Items	Post COVID-19 condition (n = 1,510)				
	Baseline		3 months		P-value*
	n	%	n	%	
Symptom(s) limit your walking?					0.221
Yes	138	9.10%	155	10.30%	
No	1,372	90.90%	1,355	89.70%	
Symptom(s) limit you in bathing/cleaning your body or dressing yourself?					0.217
Yes	30	2.00%	22	1.50%	
No	1,480	98.00%	1,488	98.50%	
Limit you in doing your usual activities such as doing housework, participating in family or leisure activities?					0.676
Yes	341	22.60%	333	22.10%	
No	1,169	77.40%	1,177	77.90%	
Can you live alone without any assistance from another person?					0.363
Yes	1,407	93.20%	1,395	92.40%	
No	103	6.80%	115	7.60%	
Symptom(s) cause negative emotions like sadness, anxiety or depression?					<0.001
Yes	434	28.70%	511	33.80%	
No	1,076	71.30%	999	66.20%	
Work affected?					0.011
Work not affected	862	57.10%	816	54.00%	
Yes, affected but still able to work	536	35.50%	584	38.70%	
Yes, lost the work or had to change job	9	0.60%	19	1.30%	
NA	103	6.80%	91	6.00%	

*McNemar’s test

Abbreviation: NA, not applicable

## Discussion

In this study, we found that 3.4% of study participants fulfilled the criteria for post COVID-19 condition during a period where SARS-CoV-2 infections in Malaysia and globally were of Omicron variant predominance. Our final analysis, however, utilised data up to 3 months only following SARS-CoV-2 infection as by this duration, the attrition rate of participants’ responses had reached 64.5% from baseline. This was partly attributed to the declining usage of the *MySejahtera* app by the public from May 2022 onwards, as registering check-ins to physical premises (one of the app’s core functions) was no longer mandatory nationally [27, 33]. Nevertheless, in our final cohort of 44,386 participants, there was a fair distribution across all groups in the socio-demographic and COVID-19 characteristics, and the characteristics were largely similar to the overall patients recruited at baseline (Table 1).

Similar studies elsewhere have reported varying findings on the prevalence of this condition. Compared to earlier studies, recent studies that included cases from Omicron predominant periods have reported lower estimated rates of post COVID-19 condition [20, 34, 35]. A previous study conducted in Malaysia involving 732 COVID-19 survivors in the community before the emergence of Omicron reported a higher prevalence rate of 21.1% [10]. The observed lower rate of 3.4% in our study can be explained in part by the decreased likelihood of post COVID-19 condition during periods when the Omicron variant predominated [20, 34, 36], as well as amongst those who are vaccinated [2, 22, 23]. Our study also followed the recent WHO definition for post COVID-19 condition [19], which may predate some of the earlier studies. It is prudent to consider factors such as the varying definitions and methods used to estimate the prevalence, varying length of follow-up periods involved, the predominant variant in circulation, as well as the different healthcare settings or populations included in the studies [7, 21, 37, 38] when comparing rates and findings across studies.

Although the estimated rates in studies following the Omicron wave appear to be lower, it is crucial to note the number of confirmed COVID-19 cases following the Omicron wave has exceeded that of all previous variants [36]. Therefore, an overall high burden in the number of people to be affected by the post COVID-19 condition is possible. To illustrate an example, as of November 2022, the UK COVID-19 Infection Survey Data estimated that 2.1 million people living in private households in the UK (3.3% of the population) to be experiencing self-reported long COVID symptoms for more than four weeks following infection [39]. In Malaysia, although over five million confirmed COVID-19 cases have been documented by 2022 [28], the total number of actual infections may likely be higher [40].

Next, our study embarked on describing the persistent symptoms experienced and identifying groups at higher risk for the post COVID-19 condition. The persistent symptoms involving multiple organ systems that are self-reported by the participants in this study are consistent with findings by numerous studies and reviews elsewhere [1, 2, 4–11]. A salient observation in our study was that an overwhelming proportion of the participants (83.8%, or more than four in five participants) reported at least one of the three most common symptoms–cough, fatigue and forgetfulness. Given the long list of related symptoms and the absence of a gold standard for evaluation and diagnosis of post COVID-19 condition, the assessment of these three symptoms may be useful to be incorporated in early screening initiatives in the future, as well as aiding efforts in risk communication and health education for the public. Further research in this aspect should be encouraged.

Our study also looked into identifying groups at higher risk of developing post COVID-19 condition and found several significant factors. Both the presence of symptoms and requiring hospital admission during the acute phase of illness (when participants were first diagnosed positive for COVID-19) had been observed to be associated with higher likelihoods of developing the post COVID-19 condition. This is consistent with findings from existing studies, where the presence (and number) of symptoms and severity of COVID-19 have been implicated [10, 15, 20, 41–44]. The higher odds observed in this study for being symptomatic, as compared to hospital admission, suggests that many who experienced mild COVID-19 may still experience persistent symptoms for up to three months duration.

We found that female participants had higher odds for post COVID-19 condition, which is consistent with findings elsewhere [10, 15, 34, 35, 44]. Differences in immune system function and sex hormones contributing to asymmetry in risk outcomes between sexes may be important drivers underlying the sex differences in post COVID-19 condition [45–47]. Although previous studies have observed that the condition increased with age [34, 41, 44], our observation for age in this study was not consistent. Although those who were aged 30–59 years old had higher odds compared to 18–29 years old, the elderly group aged 60 years and above were observed to have lower odds. This could have been due to the lower participation of the elderly group of participants, as most of the participants in this study were in the younger age groups.

Malaysia has a high NCD burden, with an estimated 1 in 3 adults living with hypertension, 1 in 5 adults living with diabetes and nearly half of the adults are overweight and obese [29, 30]. Our study cohort may be mostly in younger age groups with fewer comorbidities, but the finding that the presence of comorbidities increases the chance for post COVID-19 condition calls for concern given the high NCD burden amongst the general population in Malaysia. Similarly, other countries with significant NCD burdens should also be forewarned. This association between comorbidities and the post COVID-19 condition has also been reported in other studies [18, 35].

Identifying groups, or risk factors that increase the likelihoods of developing post COVID-19 condition is crucial to guide efforts in managing the condition, particularly in risk stratification as well as resources planning and allocation. In particular, findings from this study which was conducted in a population setting will be useful to provide a better overall picture of risk factors in the general public, compared to studies which are conducted among hospitalized or care-seeking populations as they may overrepresent those with comorbidities or at more severe spectrum of COVID-19.

In the final section of our study questionnaire, we evaluated the impact of this condition on the health-related quality of life. Notably, we found that at least one in five participants who were affected by post COVID-19 condition had limitations in performing usual daily activities, one in three had negative emotions such as sadness, anxiety and depression and around 40% of the participants reported that their work was affected. In another local study, a third of the participants who were COVID-19 survivors reported that their work performance was affected [10]. The impact on work and productivity may eventually lead to economic implications, especially since studies have reported that symptoms may continue to be present one to two years after COVID-19 diagnosis [12, 48–50].

Our study has several limitations. First, data collection was performed via a self-reported online questionnaire administered via a mobile application and therefore an objective assessment of the participants’ condition was not carried out. We were unable to conclusively rule out any alternative diagnosis which may otherwise explain the symptoms that were present. Second, as the majority of the participants in this study were at least fully vaccinated against COVID-19, we could not investigate the effect of vaccination on the post COVID-19 condition. Earlier studies have demonstrated that receipt of vaccines was associated with reduced risk of this condition [22, 23].

Overall, findings from this study underscore the gravity and impact of post COVID-19 condition as an important public health problem as we emerge from the COVID-19 pandemic; requiring strategic and responsive actions. As the symptoms cut across multiple organ systems, management of individuals affected by this condition will likely require, or benefit from, dynamic and coordinated cross-sectoral interventions involving multiple specialties. Additionally, previous studies have also highlighted that efforts and services should be all-encompassing, catering for physical or biological, psychological or experiential, social or environmental, informational as well as spiritual needs of the patients [13, 51]. In this regard, the living guidance for clinical management of COVID-19 by the WHO [52] provides recommendations and guidance for a comprehensive and holistic management plan to safeguard optimal and continuing care for COVID-19 patients. In Malaysia, efforts are ongoing–this includes putting in place a multidisciplinary management protocol for post COVID-19 patients [53] as well as rehabilitation framework models for both inpatient and outpatient services [54].

## Supporting information

S1 File Study questionnaire at baseline. (DOCX)

S2 File Clinical staging and criteria for hospital admission. (DOCX)

## References

[1] Davis HE, Assaf GS, McCorkell L, Wei H, Low RJ, Re’em Y, et al. Characterizing long COVID in an international cohort: 7 months of symptoms and their impact. EClinicalMedicine. 2021;38:101019. doi: 10.1016/j.eclinm.2021.101019 34308300 PMC8280690

[2] Davis HE, McCorkell L, Vogel JM, Topol EJ. Long COVID: major findings, mechanisms and recommendations. Nat Rev Microbiol. 2023:1–14. doi: 10.1038/s41579-022-00846-2 36639608 PMC9839201

[3] World Health Organization. Post COVID-19 condition (Long COVID) [updated 7 December 2022]. 2022 [cited 14 Apr 2023]. Available from: https://www.who.int/europe/news-room/fact-sheets/item/post-covid-19-condition

[4] Almas T, Malik J, Alsubai AK, Zaidi SM, Iqbal R, Khan K, et al. Post-acute COVID-19 syndrome and its prolonged effects: An updated systematic review. Ann Med Surg (Lond). 2022:103995. doi: 10.1016/j.amsu.2022.103995 35721785 PMC9197790

[5] Crook H, Raza S, Nowell J, Young M, Edison P. Long covid—mechanisms, risk factors, and management. BMJ. 2021;374. doi: 10.1136/bmj.n1648 34312178

[6] Groff D, Sun A, Ssentongo AE, Ba DM, Parsons N, Poudel GR, et al. Short-term and long-term rates of postacute sequelae of SARS-CoV-2 infection: a systematic review. JAMA Netw Open. 2021;4(10):e2128568. doi: 10.1001/jamanetworkopen.2021.28568 34643720 PMC8515212

[7] Healey Q, Sheikh A, Daines L, Vasileiou E. Symptoms and signs of long COVID: A rapid review and meta-analysis. J Glob Health. 2022;12:05014. doi: 10.7189/jogh.12.05014 35596571 PMC9125197

[8] Lopez-Leon S, Wegman-Ostrosky T, Perelman C, Sepulveda R, Rebolledo PA, Cuapio A, et al. More than 50 long-term effects of COVID-19: a systematic review and meta-analysis. Sci Rep. 2021;11:16144. doi: 10.1038/s41598-021-95565-8 34373540 PMC8352980

[9] Michelen M, Manoharan L, Elkheir N, Cheng V, Dagens A, Hastie C, et al. Characterising long COVID: a living systematic review. BMJ Glob Health. 2021;6(9):e005427. doi: 10.1136/bmjgh-2021-005427 34580069 PMC8478580

[10] Moy FM, Hairi NN, Lim ERJ, A Bulgiba. Long COVID and its associated factors among COVID survivors in the community from a middle-income country—An online cross-sectional study. PLoS One. 2022;17(8):e0273364. doi: 10.1371/journal.pone.0273364 36040960 PMC9426885

[11] Willi S, Lüthold R, Hunt A, Hänggi NV, Sejdiu D, Scaff C, et al. COVID-19 sequelae in adults aged less than 50 years: a systematic review. Travel Med Infect Dis. 2021;40:101995. doi: 10.1016/j.tmaid.2021.101995 33631340 PMC7898978

[12] Evans RA, Leavy OC, Richardson M, Elneima O, McAuley HJ, Shikotra A, et al. Clinical characteristics with inflammation profiling of long COVID and association with 1-year recovery following hospitalisation in the UK: a prospective observational study. Lancet Respir Med. 2022;10:761–775. doi: 10.1016/S2213-2600(22)00127-8 35472304 PMC9034855

[13] Paterson C, Davis D, Roche M, Bissett B, Roberts C, Turner M, et al. What are the long‐term holistic health consequences of COVID‐19 among survivors? An umbrella systematic review. J Med Virol. 2022;94(12):5653–5668. doi: 10.1002/jmv.28086 36002399 PMC9539336

[14] Verveen A, Wynberg E, van Willigen HD, Davidovich U, Lok A, Moll van Charante EP, et al. Health-related quality of life among persons with initial mild, moderate, and severe or critical COVID-19 at 1 and 12 months after infection: a prospective cohort study. BMC Med. 2022;20(1):1–12. doi: 10.1186/s12916-022-02615-7 36324167 PMC9629769

[15] Dryden M, Mudara C, Vika C, et al. Post-COVID-19 condition 3 months after hospitalisation with SARS-CoV-2 in South Africa: a prospective cohort study. Lancet Glob Health. 2022;10(9):e1247–e1256. doi: 10.1016/S2214-109X(22)00286-8 35961348 PMC9363040

[16] Nalbandian A, Sehgal K, Gupta A, Madhavan MV, McGroder C, Stevens JS, et al. Post-acute COVID-19 syndrome. Nat Med. 2021;27(4):601–615. doi: 10.1038/s41591-021-01283-z 33753937 PMC8893149

[17] National Institute for Health Care Excellence. COVID-19 rapid guideline: managing the long-term effects of COVID-19. (updated 11 November 2021). 2020 [cited 14 Apr 2023]. Available from: https://www.nice.org.uk/guidance/ng188

[18] Subramanian A, Nirantharakumar K, Hughes S, Myles P, Williams T, Gokhale KM, et al. Symptoms and risk factors for long COVID in non-hospitalized adults. Nat Med. 2022;28(8):1706–1714. doi: 10.1038/s41591-022-01909-w 35879616 PMC9388369

[19] World Health Organization. A clinical case definition of post COVID-19 condition by a Delphi consensus, 6 October 2021. 2021 [cited 14 Apr 2023]. Available from: https://www.who.int/publications/i/item/WHO-2019-nCoV-Post_COVID-19_condition-Clinical_case_definition-2021.1 doi: 10.1016/S1473-3099(21)00703-9 34951953 PMC8691845

[20] Arjun M, Singh AK, Roy P, Ravichandran M, Mandal S, Pal D, et al. Long COVID following Omicron wave in Eastern India-A retrospective cohort study. J Med Virol. 2022:1–8. doi: 10.1002/jmv.28214 36224705 PMC9874641

[21] Chen C, Haupert SR, Zimmermann L, Shi X, Fritsche LG, Mukherjee B. Global Prevalence of Post COVID-19 Condition or Long COVID: A Meta-Analysis and Systematic Review. J Infect Dis. 2022;226:1593–1607. doi: 10.1093/infdis/jiac136 35429399 PMC9047189

[22] Al-Aly Z, Bowe B, Xie Y. Long COVID after breakthrough SARS-CoV-2 infection. Nat Med. 2022;28(7):1461–1467. doi: 10.1038/s41591-022-01840-0 35614233 PMC9307472

[23] Ayoubkhani D, Bosworth ML, King S, Pouwels KB, Glickman M, Nafilyan V, et al. Risk of long COVID in people infected with severe acute respiratory syndrome coronavirus 2 after 2 doses of a coronavirus disease 2019 vaccine: community-based, matched cohort study. Open Forum Infect Dis. 2022;9(9):ofac464. doi: 10.1093/ofid/ofac464 36168555 PMC9494414

[24] Department of Statistics Malaysia. Demographic Statistics Third Quarter 2022, Malaysia. 2022 [cited 14 Apr 2023]. Available from: https://dosm.gov.my/portal-main/release-content/demographic-statistics-third-quarter-2022-malaysia

[25] Suah JL, Tng BH, Tok PSK, Husin M, Thevananthan T, Peariasamy KM, et al. Real-world effectiveness of homologous and heterologous BNT162b2, CoronaVac, and AZD1222 booster vaccination against Delta and Omicron SARS-CoV-2 infection. Emerg Microbes Infect. 2022;11(1):1343–1345. doi: 10.1080/22221751.2022.2072773 35499301 PMC9132393

[26] Ministry of Health Malaysia. Gelombang Varian Omicron. 2022 [cited 14 Apr 2023]. Available from: https://covid-19.moh.gov.my/semasa-kkm/2022/02/gelombang-varian-omicron

[27] Ministry of Health Malaysia. Open data on COVID-19 in Malaysia. [cited 14 Apr 2023]. Available from: https://github.com/MoH-Malaysia/covid19-public

[28] Ministry of Health Malaysia. KKMNOW: The latest data on the pandemic in Malaysia. [cited 14 Apr 2023]. Available from: https://data.moh.gov.my/covid

[29] Institute for Public Health. National Health and Morbidity Survey (NHMS) 2019: Non-communicable diseases, healthcare demand, and health literacy—Key Findings. 2020 [cited 14 Apr 2023]. Available from: https://iptk.moh.gov.my/images/technical_report/2020/4_Infographic_Booklet_NHMS_2019_-_English.pdf

[30] World Health Organization. The annual health-care cost of cardiovascular diseases, diabetes and cancer in Malaysia exceeds RM 9.65 billion. 2022 [cited 14 Apr 2023]. Available from: https://www.who.int/malaysia/news/detail/09-08-2022-the-annual-health-care-cost-of-cardiovascular-diseases—diabetes-and-cancer-in-malaysia-exceeds-rm-9.65-billion

[31] Ministry of Health Malaysia. MySejahtera. 2022 [cited 14 Apr 2023]. Available from: https://mysejahtera.moh.gov.my/en/about-mysejahtera/privacy-policy

[32] Ministry of Health Malaysia. COVID-19 Management Guidelines in Malaysia No. 5/2020. [cited 14 Apr 2023]. Available from: https://covid-19.moh.gov.my/garis-panduan/garis-panduan-kkm

[33] Ministry of Health Malaysia. MySejahtera check-in is no longer required to enter premises from 1 May 2022 [cited 14 Apr 2023]. Available from: https://covid-19.moh.gov.my/reopeningsafely/semasa/2022/04/daftar-masuk-mysejahtera-tidak-lagi-diperlukan-mulai-1-mei-2022

[34] Perlis RH, Santillana M, Ognyanova K, Safarpour A, Trujillo KL, Simonson MD, et al. Prevalence and correlates of long COVID symptoms among US adults. JAMA Netw Open. 2022;5(10):e2238804–e. doi: 10.1001/jamanetworkopen.2022.38804 36301542 PMC9614581

[35] Robertson MM, Qasmieh SA, Kulkarni SG, Teasdale CA, Jones HE, McNairy M, et al. The epidemiology of long COVID in US adults. Clin Infect Dis. 2022;ciac961. doi: 10.1093/cid/ciac961 36542514

[36] Antonelli M, Pujol JC, Spector TD, Ourselin S, Steves CJ. Risk of long COVID associated with delta versus omicron variants of SARS-CoV-2. Lancet. 2022;399(10343):2263–4. doi: 10.1016/S0140-6736(22)00941-2 35717982 PMC9212672

[37] Alkodaymi MS, Omrani OA, Fawzy NA, Abou Shaar B, Almamlouk R, Riaz M, et al. Prevalence of post-acute COVID-19 syndrome symptoms at different follow-up periods: A systematic review and meta-analysis. Clin Microbiol Infect. 2022;28(5):657–666. doi: 10.1016/j.cmi.2022.01.014 35124265 PMC8812092

[38] Woodrow MC, Carey C, Ziauddeen N, Thomas R, Akrami A, Lutje V, et al. Systematic review of the prevalence of Long Covid. Open Forum Infect Dis. 2023;10(7):ofad233. doi: 10.1093/ofid/ofad233 37404951 PMC10316694

[39] Ayoubkhani D, Sasha K. Prevalence of ongoing symptoms following coronavirus (COVID-19) infection in the UK: 3 November 2022 [cited 14 Apr 2023]. Available at: https://www.ons.gov.uk/peoplepopulationandcommunity/healthandsocialcare/conditionsanddiseases/bulletins/prevalenceofongoingsymptomsfollowingcoronaviruscovid19infectionintheuk/3november2022

[40] Jayaraj VJ, Ng C-W, Bulgiba A, Appannan MR, Rampal S. Estimating the infection burden of COVID-19 in Malaysia. PLoS Negl Trop Dis. 2022;16(11):e0010887. doi: 10.1371/journal.pntd.0010887 36346816 PMC9642899

[41] Bovil T, Wester CT, Scheel-Hincke LL, Andersen-Ranberg K. Risk factors of post-COVID-19 condition attributed to COVID-19 disease in people aged ≥50 years in Europe and Israel. Public Health. 2022;214:69–72. doi: 10.1016/j.puhe.2022.09.017 36521274 PMC9513332

[42] Ko ACS, Candellier A, Mercier M, Joseph C, Schmit JL, Lanoix JP, et al. Number of initial symptoms is more related to long COVID-19 than acute severity of infection: A prospective cohort of hospitalized patients. Int J Infect Dis. 2022;118:220–223. doi: 10.1016/j.ijid.2022.03.006 35257903 PMC8896858

[43] Silverberg JI, Zyskind I, Naiditch H, Zimmerman J, Glatt AE, Pinter A, et al. Predictors of chronic COVID-19 symptoms in a community-based cohort of adults. PloS One. 2022;17(8):e0271310. doi: 10.1371/journal.pone.0271310 35925904 PMC9352033

[44] Sudre CH, Murray B, Varsavsky T, Graham MS, Penfold RS, Bowyer RC, et al. Attributes and predictors of long COVID. Nat Med. 2021;27(4):626–631. doi: 10.1038/s41591-021-01292-y 33692530 PMC7611399

[45] Sharma G, Volgman AS, Michos ED. Sex differences in mortality from COVID-19 pandemic: are men vulnerable and women protected? JACC Case Rep. 2020;2(9):1407–1410. doi: 10.1016/j.jaccas.2020.04.027 32373791 PMC7198137

[46] Stewart S, Newson L, Briggs TA, Grammatopoulos D, Young L, Gill P. Long COVID risk-a signal to address sex hormones and women’s health. Lancet Reg Health Eur. 2021;11:100242. doi: 10.1016/j.lanepe.2021.100242 34746909 PMC8561426

[47] Sylvester SV, Rusu R, Chan B, Bellows M, O’Keefe C, Nicholson S. Sex differences in sequelae from COVID-19 infection and in long COVID syndrome: a review. Curr Med Res Opin. 2022;38(8):1391–1399. doi: 10.1080/03007995.2022.2081454 35726132

[48] Millet C, Narvaneni S, Roman S, Horani G, Chaudhry S, Michael P, et al. Symptoms persist in patients two years after COVID-19 infection: a prospective follow-up study. Clin Microbiol Infect. 2022;28(11):1505–1507. doi: 10.1016/j.cmi.2022.06.008 35724867 PMC9212679

[49] Mizrahi B, Sudry T, Flaks-Manov N, Yehezkelli Y, Kalkstein N, Akiva P, et al. Long covid outcomes at one year after mild SARS-CoV-2 infection: nationwide cohort study. BMJ. 2023;380:e072529. doi: 10.1136/bmj-2022-072529 36631153 PMC9832503

[50] Zhang X, Wang F, Shen Y, Zhang X, Cen Y, Wang B, et al. Symptoms and health outcomes among survivors of COVID-19 infection 1 year after discharge from hospitals in Wuhan, China. JAMA Netw Open. 2021;4(9):e2127403–e. doi: 10.1001/jamanetworkopen.2021.27403 34586367 PMC8482055

[51] Saunders C, Sperling S, Bendstrup E. A new paradigm is needed to explain long COVID. Lancet Respir Med. 2023;11(2):e12–e13. doi: 10.1016/S2213-2600(22)00501-X 36620963

[52] World Health Organization. Living Guidance for Clinical Management of COVID-19: Rehabilitation of adults with post COVID-19 condition. 13 Jan 2023 [cited 14 Apr 2023]. Available from: https://www.who.int/teams/health-care-readiness/post-covid-19-condition

[53] Ministry of Health Malaysia. Post COVID-19 Management Protocol (1st Edition). 2021 [cited 14 Apr 2023]. Available from: https://covid-19.moh.gov.my/garis-panduan/garis-panduan-kkm/ANNEX_50_POST_COVID-19_MANAGEMENT_PROTOCOL_12JULY2021.pdf

[54] Zamli AH. Interventions for Rehabilitation of Post COVID-19 Condition. 2021 [cited 14 Apr 2023]. Available from: https://cdn.who.int/media/docs/default-source/health-care-readiness—post-covid-19-condition/malaysia-interventions-for-rehab-post-covid-19-conditionfbd8ef9f-5800-4e9e-a949-324844f62744.pdf?sfvrsn=c011b35a_5

