# Supplementary material for: Post COVID-19 condition among adults in Malaysia following the Omicron wave: A prospective cohort study
Source: PLoS One. 2024 Jan 5;19(1):e0296488. doi: 10.1371/journal.pone.0296488 (PMC10769055; doi:10.1371/journal.pone.0296488)
Supplement: S1 File — (DOCX) [file pone.0296488.s001.docx]

**S1 FILE: STUDY QUESTIONNAIRE AT BASELINE**

**(administered seven days after the date of the positive test for SARS-CoV-2 infection)**

| Section 1 | | |
| --- | --- | --- |
| 1.1 | Sex  *Jantina* | - Male   *Lelaki*   - Female   *Perempuan* |
| 1.2 | Date of birth  *Tarikh Lahir* | dd/mm/yyyy |
| 1.3 | Race  *Kaum* | - Malay   *Melayu*   - Chinese   *Cina*   - Indian   *India*   - Others   *Lain-lain* |
| 1.4 | Employment status  *Status pekerjaan* | - Permanent job   *Pekerjaan Tetap*   - Temporary job or contract   *Pekerjaan sementara atau kontrak*   - Unemployed   *Tidak bekerja* |
| 1.5 | Highest education level  *Tahap pendidikan tertinggi* | - Primary school   *Sekolah rendah*   - Secondary School   *Sekolah menengah*   - College/University   *Kolej/Universiti*   - None   *Tiada* |
| 1.6 | Smoking currently?  *Merokok sekarang?* | - Yes   *Ya*   - No   *Tidak* |
| 1.7 | Do you have the following illness?  (May choose more than 1 option)  *Adakah anda mempunyai penyakit berikut?*  *(Boleh tanda lebih daripada 1 pilihan)* | - Diabetes   *Kencing manis*   - High blood pressure   *Darah Tinggi*   - Heart disease   *Penyakit jantung*   - Kidney disease   *Penyakit buah pinggang*   - Chronic Lung disease (i.e., asthma)   *Penyakit paru-paru kronik (seperti asma)*   - Obesity (BMI >30)   *Obesiti BMI>30*   - Cancer   *Kanser* |

| Section 2 | | | |  |
| --- | --- | --- | --- | --- |
| Do you have the following COVID-19 related symptoms now?  *Pada ketika ini, adakah anda mempunyai gejala-gejala COVID-19 yang berikut?* | | | | |
|  | Symptom  (If unsure, select “No”)  *Gejala*  *(Jika anda tidak pasti, pilih “Tiada”)* | Yes / *Ya* | No / *Tiada* | Date of onset /  Tarikh mula mengalami gejala  *Only answer this part if they answer ‘yes’ for the symptom |
| 2.1 | Fatigue or tiredness  *Keletihan badan* |  |  | dd/mm/yyyy |
| 2.2 | Muscle or joint pain  *Sakit otot atau sakit sendi* |  |  | dd/mm/yyyy |
| 2.3 | Muscle weakness  *Kelemahan otot* |  |  | dd/mm/yyyy |
| 2.4 | Breathless after physical activities  *Sesak nafas selepas melakukan aktiviti fizikal* |  |  | dd/mm/yyyy |
| 2.5 | Chest pain  *Sakit dada* |  |  | dd/mm/yyyy |
| 2.6 | Cough  *Batuk* |  |  | dd/mm/yyyy |
| 2.7 | Headache  *Sakit kepala* |  |  | dd/mm/yyyy |
| 2.8 | Sleep difficulties (insomnia)  *Sukar untuk tidur (insomnia)* |  |  | dd/mm/yyyy |
| 2.9 | Feeling anxious  *Rasa cemas* |  |  | dd/mm/yyyy |
| 2.10 | Feeling depressed  *Rasa murung* |  |  | dd/mm/yyyy |
| 2.11 | Difficult to focus  *Sukar untuk menumpu perhatian* |  |  | dd/mm/yyyy |
| 2.12 | Memory issue (forgetfulness)  *Masalah ingatan (sukar untuk ingat)* |  |  | dd/mm/yyyy |
| 2.13 | Dizziness upon standing  *Pening semasa berdiri* |  |  | dd/mm/yyyy |
| 2.14 | Loss of smell  Kehilangan bau |  |  | dd/mm/yyyy |
| 2.15 | Loss of taste  *Kehilangan rasa* |  |  | dd/mm/yyyy |
| 2.16 | Loss of appetite  *Tiada selera makan* |  |  | dd/mm/yyyy |
| 2.17 | Diarrhea  *Cirit- birit* |  |  | dd/mm/yyyy |
| 2.18 | Stomach pain  *Sakit perut* |  |  | dd/mm/yyyy |
| 2.19 | Skin rash  *Ruam kulit* |  |  | dd/mm/yyyy |
| 2.20 | Palpitation  *Jantung berdebar* |  |  | dd/mm/yyyy |

| Section 3 | | |
| --- | --- | --- |
| 3.1 | Does the above symptom(s) limit your walking?  *Adakah gejala-gejala di atas membatasi keupayaan anda untuk berjalan?* | - Yes   *Ya*   - No   *Tidak* |
| 3.2 | Does the above symptom(s) limit you in bathing/cleaning your body or dressing yourself?  *Adakah gejala-gejala di atas membatasi anda untuk mandi/membersihkan diri atau memakai pakaian sendiri?* | - Yes   *Ya*   - No   *Tidak* |
| 3.3 | Does the above symptom(s) limit you in doing your usual activities such as doing housework, participating in family or leisure activities?  *Adakah gejala-gejala di atas membatasi anda untuk menjalankan aktiviti-aktiviti biasa seperti membuat kerja rumah, menjalankan akitivti-aktiviti keluarga atau riadah?* | - Yes   *Ya*   - No   *Tidak* |
| 3.4 | Can you live alone without any assistance from another person? (e.g., independently being able to eat, walk, use the toilet and manage routine daily hygiene)  *Bolehkah anda hidup bersendirian tanpa bantuan orang lain (e.g. makan, berjalan, menggunakan tandas, dan menguruskan aktiviti kebersihan harian dengan sendiri)?* | - Yes   *Ya*   - No   *Tidak* |
| 3.5 | Does the above symptom(s) cause negative emotion like sadness, anxiety or depression?  *Adakah gejala-gejala di atas menimbulkan perasaan-perasaan negatif seperti susah hati, kegelisahan dan kemurungan?* | - Yes   *Ya*   - No   *Tidak* |
| 3.6 | Does the above symptom(s) limit your ability to work?  *Adakah gejala-gejala di atas membatasi keupayaan anda untuk bekerja?* | - No. My work is not affected   *Tidak. Kerja saya tidak terjejas*   - Yes. My work is affected but I am still able to work   *Ya. Kerja saya terjejas tapi saya masih boleh bekerja*   - Yes. I lost my work or had to change my work   *Ya. Saya kehilangan kerja atau terpaksa bertukar kerja.*   - Not applicable   *Tidak berkenaan* |
