# Supplementary material for: Post COVID-19 condition among adults in Malaysia following the Omicron wave: A prospective cohort study
Source: PLoS One. 2024 Jan 5;19(1):e0296488. doi: 10.1371/journal.pone.0296488 (PMC10769055; doi:10.1371/journal.pone.0296488)
Supplement: S2 File — (DOCX) [file pone.0296488.s002.docx]

**S2 FILE: Clinical Staging and Criteria for Hospital Admission**

Guidelines for COVID-19 management in Malaysia by the Ministry of Health Malaysia is compiled and available online: <https://covid-19.moh.gov.my/garis-panduan/garis-panduan-kkm>

(accessed on 28 Feb 2023)

**Clinical staging of confirmed COVID-19 cases in adults**

Confirmed COVID-19 patients are classified into 5 categories as stated in Table 1 below. The clinical management of the patient is based on these categories.

**Table S1: Clinical Staging of COVID-19**

| **Clinical Stage** | **Disease Severity** |
| --- | --- |
| 1 | Asymptomatic |
| 2 | Symptomatic, No Pneumonia |
| 3 | Symptomatic, Pneumonia |
| 4 | Symptomatic, Pneumonia, Requiring supplemental oxygen* |
| 5 | Critically ill with or without other organ failures |

**In patients who present with hypoxia, it is important to determine if the cause is due to COVID-19 pneumonia or other causes (e.g. bronchial asthma, fluid overload and heart failure).*

Reference:

Ministry of Health Malaysia. Annex 2e: Clinical Management Of Confirmed COVID-19 Case In Adult and Paediatric. Available online: <https://covid-19.moh.gov.my/garis-panduan/garis-panduan-kkm/ANNEX-2E-CLINICAL-MANAGEMENT-OF-CONFIRMED-COVID-19-31052022.pdf> (accessed on 28 Feb 2023)

**Criteria for Hospital/PKRC Admission**

The criteria for hospital/PKRC* admission is as below:

1. Confirmed COVID-19 patients shall be admitted to the hospital/PKRC if they fulfil any of the following criteria: (for clinical staging of COVID-19 cases, category 1-5, please refer to S2 File)
2. All category 3 to 5 disease
3. Those found to be unstable after evaluation in COVID-19 assessment centres (presence of warning signs **^1^** that may warrant admission)
4. Uncontrolled comorbidity such as OKA, hypertensive emergency, unstable angina etc.
5. Immunocompromised **^2^**
6. Age < 1 years old (category 3 and above) or paediatric with significant comorbidity ^3^ (category 2 and above)
7. Pregnant mothers in category 2 moderate and above.
8. Pregnant mothers with BMI ≥35 kg/m^2^ at booking.
9. Pregnant mothers who are unvaccinated.
10. Pregnant mothers with unstable medical /obstetrics morbidities, regardless of COVID-19 category.
11. Pregnant mothers with obstetric complaints that require in patient management (e.g. hyperemesis gravidarum, per vaginal bleeding, reduced fetal movements, etc) or thromboprophylaxis.
12. Unable to perform self-care/self-monitoring and no suitable caregiver.
13. Suspected or Probable COVID-19 cases who are clinically ill.

* PKRC is a quarantine and treatment centre that treats low risk COVID-19 patients to accommodate the increasing number of COVID-19 cases.

^1^ COVID-19 Warning Signs to be considered for admission

- Fever more than 2 days
- SPO less than 95% (at rest or after exertion)
- Angina chest pain
- Dehydration / not passing urine for more than 8 hours
- Unable to ambulate without assistance
- Reduced level of consciousness

^2^ lmmunocompromising conditions and treatments include but are not limited to:

1. Active treatment for solid tumor and hematologic malignancies.
2. Receipt of solid-organ transplant and taking immunosuppressive therapy.
3. Receipt of CAR-T-cell therapy or hematopoietic cell transplant (HCT) (within 2 years of transplantation or taking immunosuppression therapy).
4. Moderate or severe primary immunodeficiency (e.g., DiGeorge syndrome, Wiskott-Aldrich syndrome).
5. Advanced or untreated HIV infection (people with HIV and CD4 cell counts <200/mm^3^, history of an AIDS-defining illness without immune reconstitution, or clinical manifestations of symptomatic HIV).
6. Active treatment with high-dose corticosteroids (i.e., ≥20 mg prednisone or equivalent per day when administered for ≥2 weeks), alkylating agents, antimetabolites, transplant-related immunosuppressive drugs, cancer chemotherapeutic agents classified as severely immunosuppressive, tumor necrosis factor (TNF) blockers, and other biologic agents that are immunosuppressive or immunomodulatory.

^3^ Current evidence suggests that children with medical complexity, with genetic, neurologic, or metabolic conditions, or with congenital heart disease can be at increased risk for severe illness from COVID-19. Children with obesity, diabetes, asthma or chronic lung disease, thalassaemia, sickle cell disease, or immunosuppression can also be at increased risk for severe illness from COVID19 (CDC 2021; NIH 2021; RCPCH 2021).

List of comorbidities for paediatrics: (If in doubt, please contact the paediatrician)

1. Immunodeficiency:
   - - Child on immunosuppressive drugs;
     - HIV with CD4 <50 or had opportunistic infections over last 6 months;
     - Primary immunodeficiency (e.g., Severe combined immunodeficiency (SCID), Chronic granulomatous disease (CGD), Bruton's disease)
2. Child with swallowing problem, impaired cough or airway clearance (e.g., Cerebral palsy)
3. Children who are life-dependent on long term ventilation (home oxygen therapy, BIPAP, CPAP, tracheostomy)
4. Obese/syndromic child/genetic syndrome/delayed development with medically complex diseases
5. Uncontrolled diabetes mellitus
6. Thalassemia with severe iron overload
7. Post kidney transplant (first 3 months or on immunosuppressant)
8. Congenital heart disease (single ventricle pathology, cyanotic heart disease not operated, symptomatic heart failure)
9. Severe persistent bronchial asthma
10. Other chronic respiratory problems: repaired congenital thoracic abnormality

- Cystic fibrosis, bronchopulmonary dysplasia, bronchiectasis, interstitial lung disease, bronchiolitis obliterans

1. Chronic gastroenterology problems: inflammatory bowel disease, child on home TPN, decompensated liver disease, active or relapsed autoimmune liver disease.

Reference:

Ministry of Health Malaysia. Annex 2: Management of Suspected, Probable and Confirmed COVID-19 Case. <https://covid-19.moh.gov.my/garis-panduan/garis-panduan-kkm/ANNEX-2-Management-of-Suspected-Probable-and-Confirmed-COVID19-05042022.pdf> (accessed on 28 Feb 2023)
